# Network analysis of kinesiophobia and fear of progression and their association with cardiac rehabilitation adherence in post-CABG patients

**DOI:** 10.3389/fcvm.2026.1902087

**Published:** 2026-09-29

**Authors:** Zhengwei Luo, Fenghui Chen, Huihui Li, Weilong Wang, Duohuan Chen, Wen Zhu, Juan Xing, Fang Liang, Jianshuang Tao

**Affiliations:** 1 Department of Nursing, Xinjiang Medical University, Urumqi, China; 2 Department of Cardiac Surgery, People’s Hospital of Xinjiang Uygur Autonomous Region, Urumqi, China; 3 Department of Cardiac Surgery, First Affiliated Hospital of Xinjiang Medical University, Urumqi, China

**Keywords:** Bayesian network, coronary artery bypass grafting, fear of progression, kinesiophobia, network analysis

## Abstract

**Background:**

Coronary heart disease is the major disease burden of cardiovascular diseases in China, and coronary artery bypass grafting (CABG) is the preferred surgical procedure for complex coronary artery lesions. Postoperative cardiac rehabilitation can improve patient outcomes; however, kinesiophobia and fear of progression are associated with lower adherence to rehabilitation. This study aimed to identify core symptoms within the fear network, explore hypothesized directional relationships among symptoms, and analyze the associations between the two types of fear symptoms and adherence to cardiac rehabilitation.

**Methods:**

We conducted a cross-sectional survey of 294 patients who underwent their first CABG at three tertiary hospitals in Urumqi, Xinjiang, between October 2025 and February 2026. Participants were assessed using the scale of adherence to cardiac rehabilitation, the scale to measure fear of activity in patients with coronary artery disease, and the fear of progression questionnaire-short form. R software was employed to construct the symptom network. Bayesian network analysis was used to construct a directed acyclic graph, and flow network analysis was applied to examine the associations between symptoms and cardiac rehabilitation adherence.

**Results:**

In the symptom network, “My body does not allow me to do certain activities even though I would like to,” “Being afraid of no longer being able to pursue hobbies,” and “Being afraid of relying on strangers for activities of daily living” were identified as central symptoms, while “Being afraid of pain” and “Having physical symptoms, e.g., rapid heartbeat, stomachache, nervousness” served as bridge symptoms. In the Bayesian network, “Being afraid of no longer being able to pursue hobbies” was located at the apex of the network. In the flow network, a total of 19 symptoms showed negative associations with cardiac rehabilitation adherence, among which “Being afraid of pain,” “I am afraid of any physical activity since I learned my heart problem,” and “My body does not allow me to do certain activities even though I would like to” exhibited strong negative correlations. The network comparison test revealed no statistically significant differences in global strength or network structure between the middle-aged and elderly groups.

**Conclusion:**

Network analysis successfully identified the central and bridge symptoms in the kinesiophobia and fear of progression network among post-CABG patients, revealed the hypothesized directional relationships among symptoms, and identified the symptoms closely associated with cardiac rehabilitation adherence. These findings provide reference clues for postoperative symptom assessment and the subsequent development of personalized interventions.

## 1 Introduction

Coronary artery bypass grafting (CABG) remains the gold standard for treating complex coronary artery disease (1), with the annual volume reaching 49,000 cases in China in 2023 (2). Cardiac rehabilitation offers multifaceted benefits, including reduced mortality and readmission rates, improved physical function, and alleviated depressive symptoms (3, 4). However, patient adherence to cardiac rehabilitation remains suboptimal (5). As a cornerstone of cardiac rehabilitation, exercise-based intervention is crucial for preventing postoperative pulmonary complications and thromboembolism (6, 7). CABG typically involves invasive procedures such as sternotomy, vessel harvesting, and chest tube insertion. Postoperative issues, including severe pain and sternal instability (8), frequently contribute to kinesiophobia, with prevalence rates ranging from 65.69% to 77.91% (9). Furthermore, despite successful surgical outcomes, patients continue to face challenges regarding long-term prognosis. The 10-year all-cause mortality rate can reach 28% (10), and the 30-day readmission rate is 14% (11). Faced with the necessity of long-term medication and the potential risk of recurrent cardiovascular events, patients often experience persistent anxiety regarding disease exacerbation, known as fear of progression (12). Excessive fear not only compromises patients' physical and mental well-being, leading to psychological issues such as anxiety and depression, but also increases readmission rates, thereby imposing a significant economic burden on families and society (13).

The fear-avoidance model posits that when individuals develop catastrophic cognitions regarding pain, physical discomfort, or potential injury, they tend to adopt avoidant behaviors to mitigate such discomfort or perceived danger. Although this maladaptive coping strategy may provide transient relief from anxiety, it ultimately leads to physical decline and restricted mobility, thereby reinforcing fear and creating a vicious cycle of “discomfort or pain → catastrophic cognition → fear → avoidance → functional disability → exacerbated fear” (14). Among the post-CABG population, patients often catastrophize early physical activity due to concerns regarding disease exacerbation and surgical site pain, which in turn triggers kinesiophobia. This psychological fear is not only associated with lower adherence to cardiac rehabilitation and physical functional decline, but also further intensifies the fear itself. Therefore, breaking this vicious cycle and effectively managing fear among CABG patients has become pivotal to improving their adherence to cardiac rehabilitation and overall quality of life.

Most previous studies on post-CABG fear have primarily relied on total scale scores, which may obscure the intricate relationships between individual fear symptoms. Network analysis, an important psychometric method, enables precise identification of central and bridge symptoms within symptom clusters through visualization models (15). Bayesian network analysis can further evaluate conditional probability dependencies among symptoms, thereby inferring hypothesized directional relationships, while flow network analysis facilitates the investigation of associations between specific symptoms and cardiac rehabilitation adherence. By integrating these methods, this study aims to identify the central symptoms within the networks of kinesiophobia and fear of progression following CABG, as well as their associations with cardiac rehabilitation adherence, providing an empirical basis for developing targeted symptom-management interventions.

## 2 Methods

### 2.1 Participants

We used convenience sampling to recruit patients who underwent CABG at three tertiary Grade A hospitals in Urumqi, Xinjiang, between October 2025 and February 2026. Inclusion criteria: (1) meeting surgical indications and undergoing CABG for the first time; (2) age ≥ 18 years; (3) participants were informed of the study purpose and relevant matters and voluntarily agreed to participate in this study. Exclusion criteria: (1) presence of other severe physical diseases or malignant tumors; (2) presence of severe cognitive, auditory, or language impairments that precluded effective cooperation in completing the questionnaire survey. A total of 294 participants were included in this study, and a simulation procedure using netSimulator from the bootnet library was conducted to validate the adequacy of the sample size (16). The results showed that the network's correlation, sensitivity, and specificity all exceeded 0.60. This suggests that the current sample size largely provides sufficient statistical power and acceptable parameter estimation robustness for the network analysis. The Ethics Committee of the People's Hospital of Xinjiang Uygur Autonomous Region approved this study (ethics approval number: KY2025102601).

### 2.2 Measurements

#### 2.2.1 Demographic and clinical characteristics

The demographic and clinical characteristics collected in this study included sex, age, duration of coronary heart disease, chronic comorbidity status, and New York Heart Association (NYHA) functional classification.

#### 2.2.2 The scale of adherence to cardiac rehabilitation, ACRS

Wen Xiaohui and colleagues developed the scale (17). This scale consists of 33 items across five dimensions: exercise adherence, medication adherence, risk factor management and smoking and alcohol cessation adherence, nutritional management adherence, and psychological management adherence. Each item is rated on a 2-point Likert scale (0–1). Items 3, 4, and 5 were reverse-scored, with total scores ranging from 0 to 33. Higher scores indicate better adherence to cardiac rehabilitation in patients with coronary heart disease. A total score <25 indicates low adherence to cardiac rehabilitation. The Cronbach's *α* coefficient for this scale in the current study was 0.82.

#### 2.2.3 The scale to measure fear of activity in patients with coronary artery disease, fact-CAD

The scale, developed by Ozyemisci-Taskiran and colleagues (18), consists of 21 items across four dimensions: fear of exercise, avoidance of injury, body perception, and role function. Each item is rated on a 5-point Likert scale (0–4). Before network estimation, items 1, 5, 9, 13, 17, 19, and 21 were reverse-scored, with total scores ranging from 0 to 84; higher scores indicate greater kinesiophobia (19). The Cronbach's *α* coefficient for this scale in the current study was 0.93.

#### 2.2.4 Fear of progression questionnaire-short form, FoP-Q-SF

Mehnert and colleagues developed this scale (20). The scale consists of 12 items across two dimensions: physical health and social family. Each item is rated on a 5-point Likert scale (1–5), with a total score ranging from 12 to 60. Higher scores indicate greater fear of progression (21), and a total score ≥34 indicates the presence of fear of progression. The Cronbach's *α* coefficient of this scale was reported as 0.89 in a post-CABG population (22). The Cronbach's *α* coefficient for this scale in the current study was 0.93, indicating good internal consistency.

### 2.3 Data collection

Two uniformly trained graduate nursing students conducted a questionnaire-based survey within 2 to 5 days after patients were transferred out of the Intensive Care Unit. Before the survey, a standardized set of instructions explained the study's purpose and significance to participants. After obtaining written informed consent from the participants, patients were guided to complete the questionnaires. For those who were unable to complete the questionnaires independently, assistance was provided in a question-and-answer format. All questionnaires were collected and reviewed on-site immediately after completion to ensure data integrity. For items with missing responses or illegible markings, researchers pointed out the specific questions on the spot, and patients were asked to supplement or reselect their answers themselves, without any hints or guidance from the researchers. Two researchers double-checked all data before entering it into the system. A total of 300 questionnaires were distributed, and 294 valid questionnaires were retrieved (245 self-administered and 49 completed with assisted interviews), resulting in an effective response rate of 98.00%.

### 2.4 Statistical analysis and network estimation

Data analysis was performed using R software (version 4.5.2), with the packages qgraph (version 1.9.8), bootnet (version 1.8), mgm (version 1.2–15), networktools (version 1.6.0), NetworkComparisonTest (version 2.2.3), glmnet (version 5.0), ggplot2 (version 4.0.3), dplyr (version 1.2.1), tidyr (version 1.3.2), bnlearn (version 5.1), igraph (version 2.3.2), Rgraphviz (version 2.56.0). For reproducibility, a fixed random seed (12345) was set for all analyses. The analytic code is available in the Supplementary Material.

We used the bootnet (version 1.8) and qgraph packages (version 1.9.8) for network estimation and visualization. We constructed a symptom network based on the Gaussian Graphical Model (GGM), using the Spearman correlation matrix as input. In the network, nodes represent specific symptoms, and edges represent partial correlation coefficients between symptoms. The Graphical Least Absolute Shrinkage and Selection Operator (GLASSO) and the Extended Bayesian Information Criterion (EBIC) were employed for model selection, with the EBIC hyperparameter set to 0.5. We assessed centrality indices, including Expected Influence (EI) and Bridge Expected Influence (BEI) (23). The mgm package (version 1.2–15) was used to calculate node predictability, quantifying the extent to which symptoms are explained by their neighboring nodes. Network accuracy and stability were evaluated using the bootnet package (version 1.8). Non-parametric bootstrapping with 1,000 resamples was performed to calculate 95% confidence intervals (CIs) for each edge weight, thereby evaluating the accuracy of edge weights (24). Subsequently, case-dropping bootstrapping with 1,000 resamples was conducted to assess the stability of centrality indices by progressively excluding samples. The correlation stability coefficient (CS-C) was calculated, with a CS-C value > 0.50 indicating good stability (25).

A Directed Acyclic Graph (DAG) was constructed using Bayesian network analysis (26). The network structure was inferred using the hill-climbing algorithm, with the Bayesian Information Criterion for Gaussian likelihood as the scoring function, coupled with 1,000 non-parametric bootstrap resamples to evaluate structural stability (27). No arcs were constrained during structure learning, and item scores were treated as continuous variables under the Gaussian assumption. An optimal threshold of 0.5 was determined using the inclusion threshold approach to filter out unstable edges and construct the averaged network model, balancing sensitivity and specificity (28). A directed arc was formally included in the final average network model when the probability of that specific direction occurring in the bootstrap samples reached or exceeded this threshold (29). Edges with higher bootstrap occurrence probabilities were visualized with thicker lines in the network plot. Furthermore, arc strengths were quantified using the Bayesian Information Criterion (BIC) score difference, which reflects the change in network goodness-of-fit upon edge removal; a larger absolute value indicates a stronger conditional dependency associated with that directed edge (30). The directions of edges represent statistical hypotheses rather than temporal causal inferences, and are intended to provide preliminary clues for future longitudinal studies and clinical interventions.

To explore the relationship between symptoms and cardiac rehabilitation adherence, the ACRS score was integrated into the network model and visualized using the flow function in the qgraph package (version 1.9.8) (31). Following the World Health Organization's age classification criteria for older adults in developing countries, we used 60 years as the cutoff to distinguish between middle-aged and elderly groups and examine differences in the fear symptom network across age groups (32). We used the NetworkComparisonTest package (version 2.2.3) to compare networks between the middle-aged group (<60 years) and the elderly group (≥60 years). Tests included network structure invariance and global strength invariance, with *P* < 0.05 indicating statistical significance (33).

## 3 Results

### 3.1 Demographic and clinical characteristics

The 294 patients were recruited from three tertiary Grade A hospitals in Urumqi, Xinjiang: the People's Hospital of Xinjiang Uygur Autonomous Region (*n* = 113), the First Affiliated Hospital of Xinjiang Medical University (*n* = 136), and the Fourth Affiliated Hospital of Xinjiang Medical University (*n* = 45). The mean age of the 294 post-CABG patients was 56.218 ± 10.316 years, with 130 (44.22%) being elderly (aged ≥60 years). There were 230 (78.23%) males and 64 (21.77%) females. Regarding clinical characteristics, 242 (82.31%) patients had a coronary heart disease duration of <5 years, and 52 (17.69%) had a duration of ≥5 years. Regarding comorbidities, 178 patients (60.54 %) had ≥2 chronic diseases, and 116 patients (39.46 %) had <2 chronic diseases. New York Heart Association (NYHA) cardiac function classification: 138 (46.94%) were Class II, 153 (52.04%) were Class III, and 3 (1.02%) were Class IV. Demographic and clinical characteristics are presented in Table 1. The mean ACRS score was 27.058 ± 4.295, with 27.89% (95% *CI*: 23.08%–33.28%) of patients exhibiting low adherence to cardiac rehabilitation. The mean FoP-Q-SF score was 27.082 ± 9.847, and 27.89% (95% *CI*: 23.08%–33.28%) of patients were identified as having fear of progression. The mean Fact-CAD score was 39.888 ± 12.951.

**Table 1:** Demographic and clinical characteristics.

Variable	Categories	*N* (%)
Age (years)	<60	164 (55.78)
	≥60	130 (44.22)
Gender	Men	230 (78.23)
	Women	64 (21.77)
Duration of coronary heart disease (years)	<5	242 (82.31)
	≥5	52 (17.69)
Chronic comorbidity status	≥2	178 (60.54)
	<2	116 (39.46)
NYHA functional classification	II	138 (46.94)
	III	153 (52.04)
	IV	3 (1.02)

### 3.2 Network structure

The symptom network structure of kinesiophobia and fear of progression among post-CABG patients is illustrated in Figure 1A. Among the 528 potential edges in the network, 233 (44.13%) were non-zero (see Supplementary Table S1). The average node predictability was 47.38%, indicating that, on average, 47.38% of the variance in each symptom can be explained by its neighboring nodes. Figure 1B shows the centrality indices. FOA4 “My body does not allow me to do certain activities even though I would like to” (EI = 1.334) exhibited the highest EI. Additionally, FOP8 “Being afraid of no longer being able to pursue hobbies” (EI = 1.153) and FOP7 “Being afraid of relying on strangers for activities of daily living” (EI = 1.111) demonstrated high levels of EI and predictability. Notably, FOA11 “I now try to protect myself from strenuous activities” (EI = −0.290) exhibited a negative EI and zero predictability. FOP3 “Being afraid of pain” (BEI = 0.334) showed the highest BEI, followed by FOP5 “Having physical symptoms, e.g., rapid heartbeat, stomachache, nervousness” (BEI = 0.332). Table 2 presents the EI, BEI, and predictability of each node in the network. Bootstrap analysis of edge weights revealed that the 95% CIs from the resampled data and the original data were narrow, indicating good accuracy of the network edge weights (see Supplementary Figure S1). The network stability analysis showed that the CS-C values for EI and BEI were 0.60 and 0.52, respectively, both exceeding 0.5, indicating good stability of the centrality indices (see Supplementary Figure S2). Bootstrap-based difference tests further evaluated whether the differences in centrality indices and edge weights were statistically significant (see Supplementary Figures S3, S4). Black cells indicate statistically significant differences between two nodes, whereas gray cells indicate non-significant differences. For EI, FOA4 “My body does not allow me to do certain activities even though I would like to” showed the highest EI, with statistically significant differences from most other nodes, suggesting that it may occupy a relatively central position in the current network; FOP8 “Being afraid of no longer being able to pursue hobbies” and FOP7 “Being afraid of relying on strangers for activities of daily living” also showed relatively high EI. However, high centrality only indicates stronger connections within the estimated cross-sectional network and should not be directly interpreted as clinical importance or causal influence. For BEI, FOP3 “Being afraid of pain” and FOP5 “Having physical symptoms, e.g., rapid heartbeat, stomachache, nervousness” exhibited relatively high BEI, with no statistically significant difference between them, suggesting that they may serve as bridge symptoms linking kinesiophobia and fear of progression.

**Figure 1:**
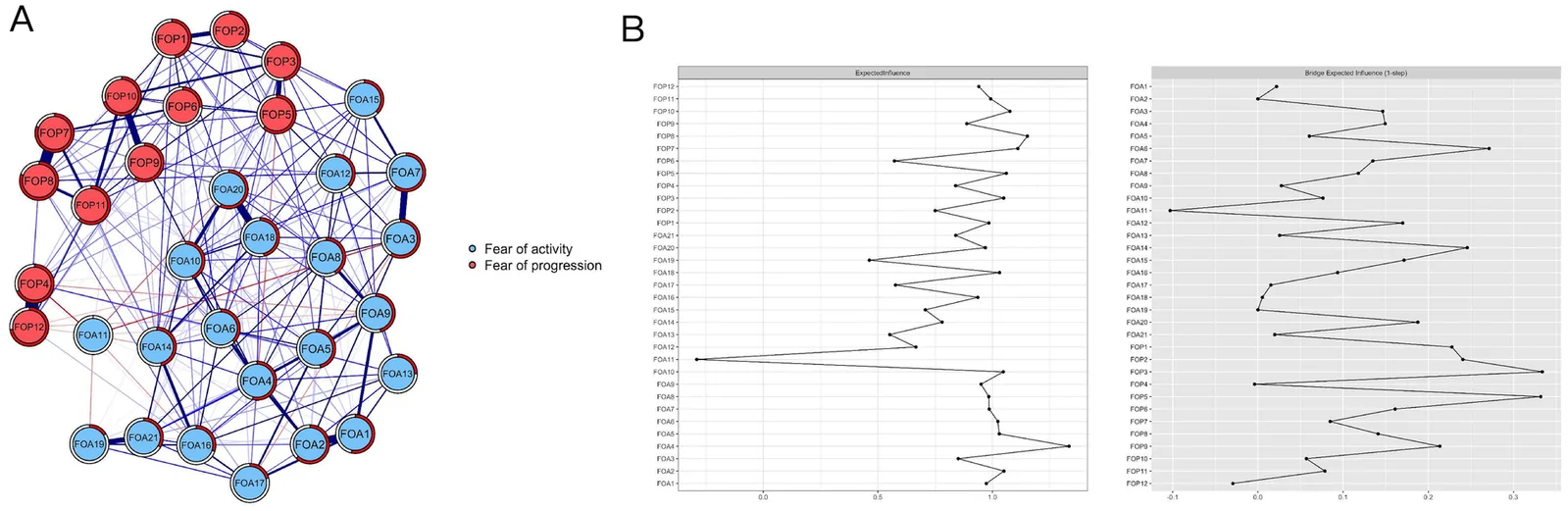
Network structure and centrality index of kinesiophobia and fear of progression symptoms. **(A)** Network graph of kinesiophobia and fear of progression in patients after CABG. **(B)** Raw scores for the centrality index of the network. Abbreviations are explained in Table 2. Blue edges indicate positive correlations; red edges indicate negative correlations. Thicker and more intense edges indicate stronger correlations.

**Table 2:** Centrality indicators and predictability of network nodes for kinesiophobia and fear of progression in patients after CABG surgery.

Items	Content	EI	BEI	Predictability
FOA1	I can walk on level road as long as I want to despite my heart problem	0.973	0.022	0.532
FOA2	I am afraid of any physical activity since I learned my heart problem	1.050	0.000	0.624
FOA3	I feel uneasy when I walk uphill due to my heart problem	0.851	0.146	0.585
FOA4	My body does not allow me to do certain activities even though I would like to	1.334	0.149	0.539
FOA5	Morning exercises relax me	1.030	0.060	0.464
FOA6	I have difficulty in performing my duties at home due to my heart problem	1.024	0.271	0.376
FOA7	I feel chest discomfort if I exert myself a little	0.986	0.135	0.599
FOA8	I cannot carry loads heavier than three kilograms after my heart problem	0.984	0.118	0.416
FOA9	I do the shopping for my home despite my heart problem	0.950	0.028	0.458
FOA10	It is not possible to maintain my usual physical activities after learning about my heart problem	1.048	0.076	0.436
FOA11	I now try to protect myself from strenuous activities	−0.290	−0.103	0.000
FOA12	I feel physically insufficient	0.666	0.170	0.366
FOA13	My sexual life is as it used to be	0.552	0.025	0.250
FOA14	I refrain from staying alone due to my heart problem	0.780	0.246	0.463
FOA15	I am worried that I may hurt my heart/incision if I exercise	0.707	0.171	0.374
FOA16	I feel demoralized to see that my spouse/relatives are more protective of me	0.936	0.094	0.302
FOA17	I will exercise if my doctors say so	0.577	0.015	0.210
FOA18	It is natural that I am unable to do certain activities that I used to do	1.031	0.005	0.467
FOA19	My heart problem may get worse if I stay inactive	0.463	0.000	0.162
FOA20	I am worried that I will not be able to be as strong as I were after my heart problem	0.969	0.188	0.575
FOA21	Being physically active makes me feel good	0.840	0.020	0.334
FOP1	Being afraid of disease progression	0.985	0.228	0.469
FOP2	Being nervous prior to doctors’ appointments or periodic examinations	0.750	0.241	0.360
FOP3	Being afraid of pain	1.049	0.334	0.474
FOP4	Being afraid of becoming less productive at work	0.839	−0.004	0.686
FOP5	Having physical symptoms, e.g., rapid heartbeat, stomachache, nervousness	1.061	0.332	0.622
FOP6	Being afraid of the possibility that the children could contract disease	0.572	0.161	0.256
FOP7	Being afraid of relying on strangers for activities of daily living	1.111	0.085	0.807
FOP8	Being afraid of no longer being able to pursue hobbies	1.153	0.141	0.806
FOP9	Being afraid of severe medical treatments in course of the illness	0.888	0.214	0.587
FOP10	Worrying that medications could damage the body	1.076	0.057	0.698
FOP11	Worrying about what will become of the family if something should happen to the patient	0.992	0.079	0.611
FOP12	Being afraid of not being able to work anymore	0.940	−0.029	0.727

EI, Expected Influence; BEI, Bridge Expected Influence. EI and BEI are raw scores.

### 3.3 Bayesian network analysis

The DAG of the Bayesian network is presented in Figure 2. The network retained 66 directed edges (see Supplementary Table S2). Edges with larger BIC differences included the edge between FOP8 and FOP7 (BIC = −239.768) and the edge between FOP4 and FOP12 (BIC = −156.598), suggesting strong conditional dependencies between these node pairs. FOP8 “Being afraid of no longer being able to pursue hobbies” was positioned at the apex of the DAG and had direct directed edges to FOP7, FOP11, FOP12, and FOA12. Notably, the majority of the fear of progression symptoms are positioned upstream of the kinesiophobia symptoms within the network. It should be emphasized that the aforementioned directional connections are statistical inferences based on the current data and model assumptions, and do not represent temporal or causal relationships among the variables.

**Figure 2:**
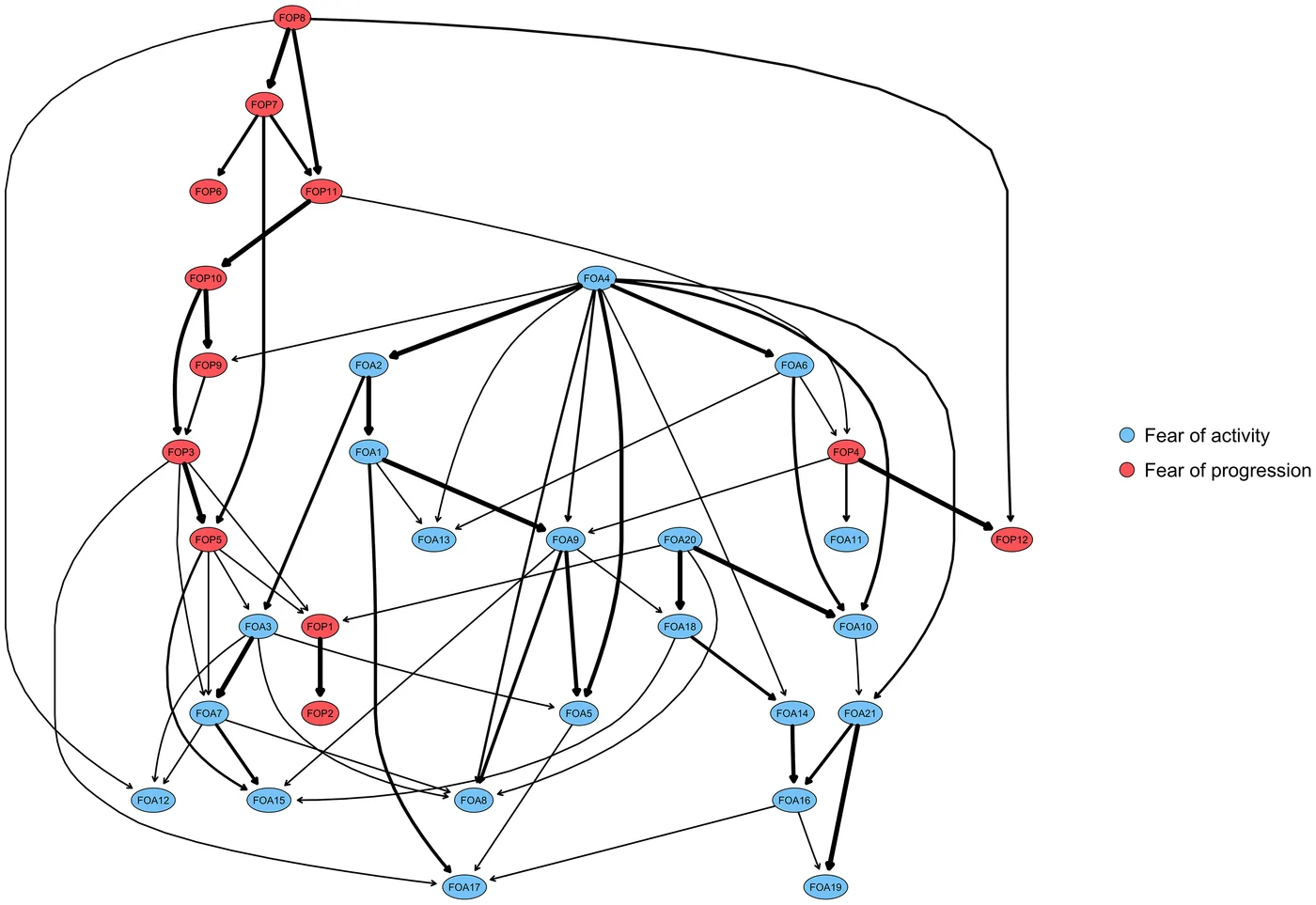
Directed acyclic graph of kinesiophobia and fear of progression in patients after CABG. Abbreviations are explained in Table 2.

### 3.4 Association between fear symptoms and adherence to cardiac rehabilitation

Flow network analysis revealed that 19 symptoms were negatively associated with adherence to cardiac rehabilitation. Among these, FOP3 “Being afraid of pain” (weight = −0.158), FOA2 “I am afraid of any physical activity since I learned my heart problem” (weight = −0.109), and FOA4 “My body does not allow me to do certain activities even though I would like to” (weight = −0.084) exhibited strong negative associations with cardiac rehabilitation adherence (see Figure 3). Supplementary Table S3 presents the edge-weight values between each symptom and cardiac rehabilitation adherence.

**Figure 3:**
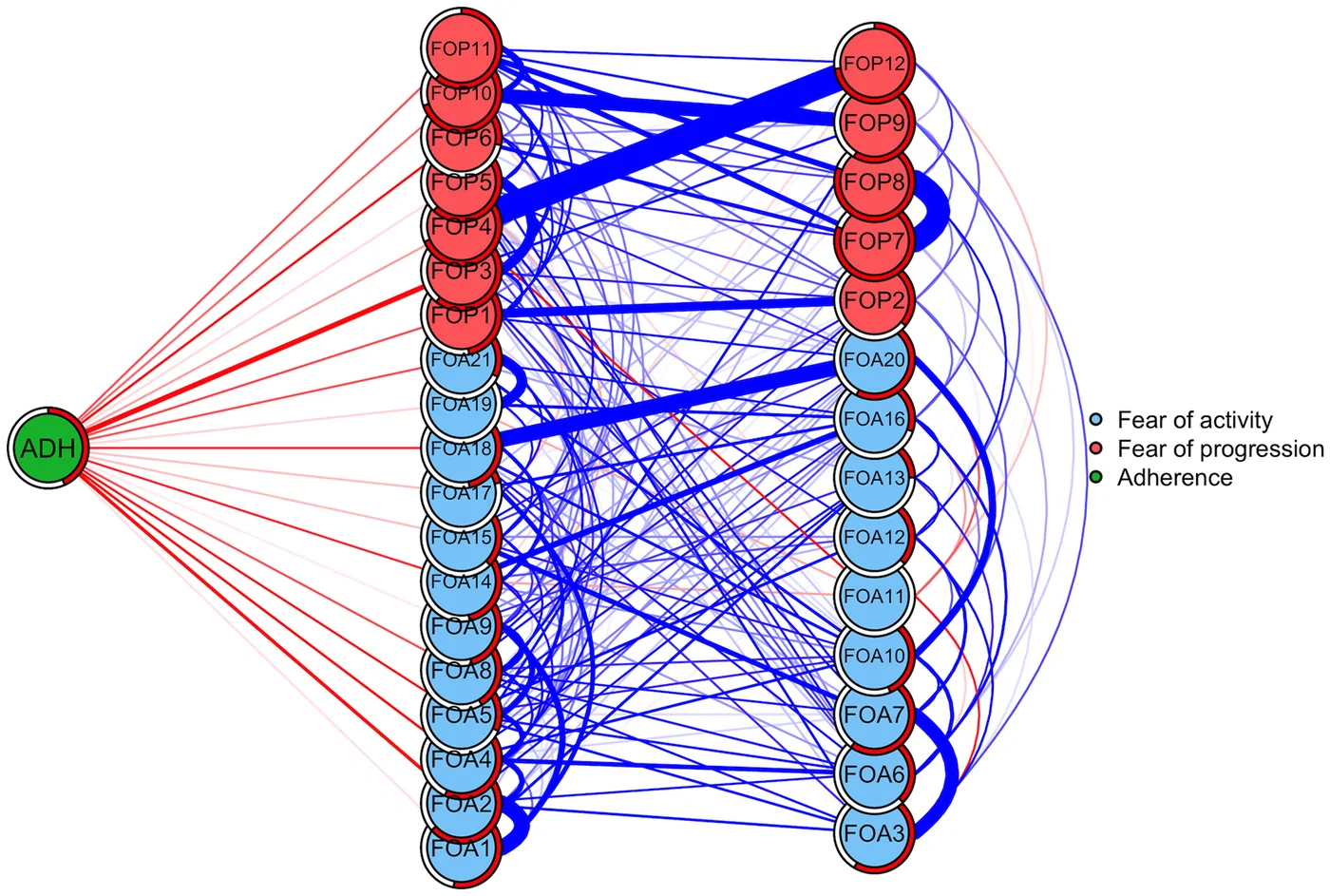
Flow network graph of kinesiophobia, fear of progression, and cardiac rehabilitation adherence in patients after CABG. Abbreviations are explained in Table 2. Blue edges indicate positive correlations; red edges indicate negative correlations. Thicker and more intense edges indicate stronger correlations.

### 3.5 Network comparison between different age groups

The network comparison test revealed no statistically significant differences in global strength (*p* = 0.357) or network structure (*p* = 0.343) between the middle-aged group (< 60 years) and the elderly group (≥60 years).

## 4 Discussion

This study used network analysis to construct a symptom network model of kinesiophobia and fear of progression in patients undergoing CABG, and combined Bayesian network and flow network analysis to identify hypothesized directional relationships among symptoms and their associations with adherence to cardiac rehabilitation. Core symptoms identified via network analysis should be regarded as key screening clues rather than direct intervention targets; establishing clinical intervention priorities still requires causal evidence from longitudinal or intervention simulation studies.

Centrality analysis showed that “My body does not allow me to do certain activities even though I would like to” exhibited the highest EI, indicating it as the central symptom of the symptom network. Factors such as significant incisional pain after CABG (34), the presence of multiple invasive catheters (35), and the general concern about adverse events may predispose patients to develop such concerns. According to the fear-avoidance model, patients tend to catastrophically interpret chest pain, sternal pulling discomfort, and the risk of cardiac reinjury, thereby amplifying the threat of minor physical discomfort. Even when their physiological condition may tolerate physical activity, they subjectively perceive their body as incapable of completing the activity (36). The traumatic experience and fatigue associated with major cardiac surgery may also undermine patients' exercise self-efficacy and reduce their confidence in their own physical capabilities (37). Previous studies have confirmed that excessive protective care from family members can impede the recovery of patients' physical activity ability. Therefore, health education should be provided to family members concurrently, guiding them to avoid overprotective restrictions (38). At the same time, patients should be encouraged to actively participate in daily self-care activities and family decision-making within safe boundaries to rebuild their self-identity and self-efficacy. Additionally, create a quiet, comfortable ward environment for patients. Medical procedures should be reasonably scheduled to minimize external stimuli; various tubes and catheters should be properly secured; and patients should be assisted in assuming comfortable positions, all of which may help alleviate postoperative physical discomfort. For patients' irrational cognitions, cognitive-behavioral therapy could be introduced to help patients recognize negative thought patterns and correct excessive worries when facing physical discomfort and the risk of disease recurrence (39, 40).

In addition, “Being afraid of no longer being able to pursue hobbies” and “Being afraid of relying on strangers for activities of daily living” exhibited high EI in the network. Consistent with Gao et al. (29), who identified “being afraid of relying on strangers for daily activities in the future” as a central symptom in the network of anxiety, depression, and fear of progression among parents of children with malignant solid tumors. Due to the substantial surgical trauma of CABG and the prolonged sternal healing period, patients require assistance from others for basic activities of daily living, such as toileting, washing, dressing, and turning over, for a considerable period postoperatively, which may lead patients to form catastrophic expectations regarding their future self-care abilities. This suggests that clinical nurses should introduce cardiac rehabilitation programs as early as possible and effectively enhance patients' confidence in recovering health and returning to society through step-by-step exercise rehabilitation plans (3). In contrast, FOA11 “I now try to protect myself from strenuous activities” exhibited a negative EI and zero predictability in the symptom network. Of the 13 retained edges of FOA11, 12 were negative (such as FOA11–FOP4, weight = −0.076; FOA11–FOA3, weight = −0.062; and FOA11–FOA20, weight = −0.041), with only one trivial positive edge (FOA11–FOA1, weight = +0.004); although these edges were retained in the regularized network, they were all weak and mixed in direction, contributing almost nothing to the explained variance of the node. This finding may be related to the clinical context of the early postoperative period: within 2 to 5 days after transfer out of the ICU, avoiding strenuous activities is a routinely followed activity recommendation for CABG patients, which may, to some extent, weaken the associations of this item with other symptoms.

Bridge centrality analysis further identified the key nodes connecting different symptom clusters, and the results showed that “Being afraid of pain” had the highest BEI, serving as a bridge symptom between the two symptom clusters of kinesiophobia and fear of progression. This is similar to the findings of the fear symptom network analysis conducted by YanRong Gu et al. among patients after percutaneous coronary intervention (41). Post-thoracotomy surgical trauma and stimulation can activate the sympathetic-adrenal medullary system, leading to a massive release of catecholamines and inducing a series of physical stress manifestations such as increased heart rate and muscle tension (42). Under this state of hypervigilance, patients are prone to misinterpreting normal physical reactions during the recovery period—such as palpitations, incision traction pain, and fatigue—as signals of disease deterioration (43), thereby reinforcing avoidance behaviors and fear of pain (44). In addition, “Having physical symptoms, e.g., rapid heartbeat, stomachache, nervousness” also exhibited a relatively high BEI. During CABG surgery, cardiopulmonary bypass can cause atrial tissue edema and electrophysiological disturbances, inducing postoperative atrial arrhythmias and palpitations (45); meanwhile, sedative medications and early postoperative mobility restrictions can inhibit gastrointestinal motility (46), leading to abdominal discomfort such as bloating and constipation (47). According to symptom network theory, the interactions between different symptom clusters are often transmitted and diffused through bridge symptoms (23). In clinical practice, it is recommended that pain and physical discomfort be incorporated into routine nursing assessments after CABG to enable early identification and dynamic monitoring. At the same time, patients may be guided to differentiate between normal postoperative recovery responses and signs of deterioration to reduce hypervigilance to physical sensations. Non-pharmacological interventions such as progressive muscle relaxation and breathing regulation may also be used as adjuncts to alleviate pain and various physical symptoms (48).

Although cross-sectional studies cannot confirm the temporal relationships among symptoms, DAGs can still provide important clues for exploring hypothesized directional relationships among symptoms (29). Bayesian network analysis revealed that “Being afraid of no longer being able to pursue hobbies” was located at the top of the DAG. This result corroborated the symptom network analysis, and this item was simultaneously identified as a central symptom of the network. This finding suggests that patients' fear about not being able to resume their previous hobbies postoperatively may occupy a central position in the interconnections among fear symptoms. Post-CABG patients often develop kinesiophobia due to the fear of inducing angina pectoris or worsening their condition, deliberately avoiding daily activities and hobbies. Based on these findings, clinical nursing can focus on patients' expectations regarding postoperative mobility and hobby recovery and, combined with individualized assessment, guide patients to gradually resume appropriate hobby activities under stable illness conditions and permitted circumstances. In addition, the Bayesian network also showed that symptoms related to fear of progression were mostly located upstream of kinesiophobia symptoms. From the perspective of probabilistic priority, it can be hypothesized that patients' cognitive appraisals of fear of progression may precede kinesiophobia or be correlated with it. This aligns with the theoretical tenets of the fear-avoidance model (14). However, longitudinal studies are needed to confirm this relationship (28).

Flow network analysis showed that 19 symptoms were negatively correlated with adherence to cardiac rehabilitation. Among them, “Being afraid of pain” exhibited a relatively strong negative correlation with cardiac rehabilitation adherence, followed by “I am afraid of any physical activity since I learned my heart problem” and “My body does not allow me to do certain activities even though I would like to.” Existing studies indicate that 74% of post-CABG patients experience moderate to severe pain postoperatively (49). Postoperative pain not only directly limits patients' activities of daily living but may also lead to a delayed initiation of the rehabilitation process (50). Existing studies point out that pain catastrophizing not only directly induces kinesiophobia but may also indirectly weaken rehabilitation adherence by reducing exercise self-efficacy (51). These findings suggest that during the initiation and advancement phases of cardiac rehabilitation, multidisciplinary collaboration with the pain department and physical therapists should be strengthened to formulate individualized analgesic regimens (52), ensuring that patients remain in a tolerable and comfortable state during rehabilitation training. Second, pain cognitive restructuring should be conducted to help patients view moderate pain during the rehabilitation process scientifically, thereby reducing their pain catastrophizing (53). Finally, graded exposure techniques can be introduced under the professional guidance of rehabilitation therapists to guide patients from low-intensity, pain-free activities to cardiac rehabilitation training, thereby enhancing participation and adherence (35).

This study has several limitations. First, the study subjects were recruited exclusively from tertiary Grade-A hospitals in Urumqi, and regional differences in medical cultures, postoperative rehabilitation education models, and social support environments may influence the symptom network (54); therefore, caution is warranted when generalizing these findings to post-CABG patients nationwide. Second, male patients accounted for 78.23% of the participants, resulting in a limited female sample size. Previous studies suggest that female patients are more prone to prominent pain experiences and higher levels of anxiety after cardiac surgery, and the symptom combinations and internal association patterns of kinesiophobia and fear of progression may differ from those of males (9, 55); thus, caution is warranted when generalizing these conclusions to female populations. In addition, although the CS-C of the network in this study was at an acceptable level, the sample size remains somewhat insufficient given the inclusion of 33 network nodes, which may affect the precision of model estimation. Moreover, the network comparison test between the middle-aged and elderly groups was based on relatively small subgroups, which limits the statistical power to detect between-group differences; accordingly, the absence of statistically significant differences in global strength or network structure should be interpreted with caution. Furthermore, the use of convenience sampling may have introduced selection bias, and patients were recruited from three tertiary hospitals, which may have induced clustering effects not accounted for in the analyses.

Meanwhile, although Bayesian networks help explore hypothesized pathways among symptoms, the cross-sectional study design limits their ability to confirm temporal relationships and dynamic evolutionary processes among variables. Questionnaires in this study were administered 2 to 5 days after patients were transferred out of the ICU, and the ACRS scale reflects patients’ early postoperative rehabilitation intention rather than their true long-term rehabilitation adherence. All variables were assessed using self-report measures, which may have introduced common method bias. Moreover, the questionnaires were administered through two modes; this administration-mode difference may have introduced additional measurement variance. Additionally, some items of the FoP-Q-SF and the Fact-CAD overlap in content, which may have strengthened the bridge connections between the two symptom clusters and inflated the bridge centrality of these nodes. In addition, the FoP-Q-SF was originally developed and validated in chronic disease populations such as cancer patients; its construct validity and the appropriateness of the cutoff (≥34) in post-CABG patients have not been specifically established, and thus the classification of fear of progression in this population warrants cautious interpretation. Furthermore, this study did not collect key post-CABG clinical indicators—such as the number of bypass grafts, postoperative pain severity, and surgical complications—which may represent unmeasured confounding factors. Finally, the network analysis did not statistically adjust for demographic and clinical covariates, posing a risk of confounding bias. Future research should conduct multicenter, large-sample studies with a balanced inclusion of a sufficient number of female participants to validate the symptom network model constructed in this study; meanwhile, longitudinal tracking designs combined with cross-lagged panel network models should be employed to explore potential underlying association mechanisms among symptoms. Incorporating covariate adjustment strategies into the network estimation process—such as treating age, sex, and duration of coronary heart disease as covariate nodes or applying regression-correction analytical methods—can reduce confounding and more accurately estimate symptom associations.

## 5 Conclusions

In summary, this study constructed a symptom network model of kinesiophobia and fear of progression in patients following CABG, identified core symptoms, and analyzed their associations with cardiac rehabilitation adherence. The results showed that “My body does not allow me to do certain activities even though I would like to,” “Being afraid of no longer being able to pursue hobbies,” and “Being afraid of relying on strangers for activities of daily living” were identified as central symptoms; “Being afraid of pain” and “Having physical symptoms, e.g., rapid heartbeat, stomachache, nervousness” served as bridge symptoms; “Being afraid of no longer being able to pursue hobbies” was located at the apex of the DAG; “Being afraid of pain”, “I am afraid of any physical activity since I learned my heart problem,” and “My body does not allow me to do certain activities even though I would like to” exhibited strong negative correlations with cardiac rehabilitation adherence. The identified symptom nodes can serve as candidate targets for subsequent longitudinal validation and intervention studies, providing reference clues for clinical nursing symptom assessment and the subsequent development of personalized intervention programs.

## Data Availability

The raw data supporting the conclusions of this article will be made available by the authors, without undue reservation.

## References

[1] Doenst T Haverich A Serruys P Bonow RO Kappetein P Falk V. PCI and CABG for treating stable coronary artery disease: jACC review topic of the week. J Am Coll Cardiol. (2019) 73:964–76. 10.1016/j.jacc.2018.11.053 30819365

[2] National Center for Cardiovascular Diseases The Writing Committee of the Report on Cardiovascular Health and Diseases in China. Report on cardiovascular health and diseases in China 2023: an updated summary. Biomed Environ Sci. (2024) 37:949–92. 10.3967/bes2024.162 39401992

[3] Huang J Liu C Xie J Han J. Cardiac rehabilitation for elderly patients undergoing coronary artery bypass grafting: a systematic review and meta-analysis. BMC Surg. (2025) 25:574. 10.1186/s12893-025-03315-8 41299466 PMC12659137

[4] Chong MS Sit JWH Choi KC Suhaimi A Chair SY. Barriers to cardiac rehabilitation and patient perceptions on the usage of technologies in cardiac rehabilitation: a cross-sectional study. J Clin Nurs. (2024) 33:1084–93. 10.1111/jocn.16919 37909483

[5] Zhang S Ding R Chen S Meng X Jianchao L Wang DW. Availability and trend of dissemination of cardiac rehabilitation in China: report from the multicenter national registration platform between 2012 and 2021. Front Cardiovasc Med. (2023) 10:1210068. 10.3389/fcvm.2023.1210068 37404729 PMC10315840

[6] Dibben GO Faulkner J Oldridge N Rees K Thompson DR Zwisler AD. Exercise-based cardiac rehabilitation for coronary heart disease: a meta-analysis. Eur Heart J. (2023) 44:452–69. 10.1093/eurheartj/ehac747 36746187 PMC9902155

[7] Xing W Piao JJ Ren T Liang Y Li Q Gu Y. Classification of kinesiophobia in patients after cardiac surgery under extracorporeal circulation in China: latent profile and influencing factors analysis from a cross-sectional study. BMJ Open. (2025) 15:e083909. 10.1136/bmjopen-2024-083909 39855652 PMC11758704

[8] Zubrzycki M Liebold A Skrabal C Reinelt H Ziegler M Perdas E. Assessment and pathophysiology of pain in cardiac surgery. J Pain Res. (2018) 11:1599–611. 10.2147/JPR.S162067 30197534 PMC6112778

[9] Zeng Z Shen Y Wan L Yang X Hu Q Luo H. Kinesiophobia in patients after cardiac surgery: a scoping review. BMC Cardiovasc Disord. (2024) 24:469. 10.1186/s12872-024-04140-2 39223455 PMC11370225

[10] Pack QR Goel K Lahr BD Greason KL Squires RW Lopez-Jimenez F. Participation in cardiac rehabilitation and survival after coronary artery bypass graft surgery: a community-based study. Circulation. (2013) 128:591–7. 10.1161/CIRCULATIONAHA.112.001365 23836837

[11] Montrief T Koyfman A Long B. Coronary artery bypass graft surgery complications: a review for emergency clinicians. Am J Emerg Med. (2018) 36:2289–97. 10.1016/j.ajem.2018.09.014 30217621

[12] Li Y Zhong X Dai P Chen B Zhou F Fan Y. Construction and validation of a risk predictive model for fear of disease progression in patients after percutaneous coronary intervention. Sci Rep. (2025) 16:1444. 10.1038/s41598-025-31099-7 41353494 PMC12796424

[13] Xu Y Ma H-X Liu S-S Gong Q. Correlation among anxiety and depression, fear of disease progression, and social support in coronary heart disease. World J Psychiatry. (2024) 14:1708–17. 10.5498/wjp.v14.i11.1708 39564166 PMC11572683

[14] Vlaeyen JWS Linton SJ. Fear-avoidance and its consequences in chronic musculoskeletal pain: a state of the art. Pain. (2000) 85:317–32. 10.1016/S0304-3959(99)00242-0 10781906

[15] Bian Z Shang B Luo C Lv F Sun W Gong Y. Exploring symptom clusters and core symptoms during the vulnerable phase in patients with chronic heart failure: a network-based analysis. Eur J Cardiovasc Nurs. (2025) 24:279–87. 10.1093/eurjcn/zvae152 39743303

[16] Epskamp S Borsboom D Fried EI. Estimating psychological networks and their accuracy: a tutorial paper. Behav Res Methods. (2018) 50:195–212. 10.3758/s13428-017-0862-1 28342071 PMC5809547

[17] Chai Z Fan Y Gong X Zhang Y Hu Y Li X. Adherence to phase I cardiac rehabilitation in post-PCI patients: a latent class analysis. Front Cardiovasc Med. (2025) 12:1460855. 10.3389/fcvm.2025.1460855 40066356 PMC11891241

[18] Ozyemisci-Taskiran O Demirsoy N Atan T Yuksel S Coskun O Aytur YK. Development and validation of a scale to measure fear of activity in patients with coronary artery disease (fact-CAD). Arch Phys Med Rehabil. (2020) 101:479–86. 10.1016/j.apmr.2019.09.001 31562874

[19] Chen J Guo W Liu H Liu W Chen J Ma C. Translation and psychometric evaluation of a scale to measure the fear of activity in Chinese patients with coronary artery disease. Nurs Open. (2023) 10:4033–43. 10.1002/nop2.1663 36840660 PMC10170931

[20] Mehnert A Herschbach P Berg P Henrich G Koch U. Progredienzangst bei brustkrebspatientinnen—validierung der kurzform des progredienzangstfragebogens PA-F-KF/ fear of progression in breast cancer patients—validation of the short form of the fear of progression questionnaire (FoP-Q-SF). Z Psychosom Med Psychother. (2006) 52:274–88. 10.13109/zptm.2006.52.3.274 17156600

[21] Liu S Zhang Y Miao Q Zhang X Jiang X Chang T. The mediating role of self-perceived burden between social support and fear of progression in renal transplant recipients: a multicenter cross-sectional study. Psychol Res Behav Manag. (2023) 16:3623–33. 10.2147/PRBM.S424844 37693331 PMC10488562

[22] Wu A Liu H Fang T Fan P Yin C Shen S. The effects of PERMA model-based positive psychological intervention on fear of disease progression and subjective well-being in patients after coronary artery bypass grafting: a retrospective cohort study. Front Cardiovasc Med. (2026) 13:1784517. 10.3389/fcvm.2026.1784517 42291561 PMC13253467

[23] Luo H Wang T. A network analysis of anxiety and depression symptoms among empty nesters in China. Front Psychol. (2025) 16:1667813. 10.3389/fpsyg.2025.1667813 41050802 PMC12488660

[24] Guo Z Li Y Wang L Lin X Sun J Yu J. Mental health concerns in patients with COVID-19: a network analysis. Psychol Res Behav Manag. (2025) 18:1125–37. 10.2147/PRBM.S524256 40406339 PMC12095440

[25] Zhang X Lin R Zhang Z Hu Q Li P Fei X. Comorbidity network of self-stigma, insomnia, and mental health in chronic disease patients: a network analysis. Psychol Res Behav Manag. (2025) 18:2333–45. 10.2147/PRBM.S529940 41341991 PMC12671085

[26] Briganti G Scutari M McNally RJ. A tutorial on Bayesian networks for psychopathology researchers. Psychol Methods. (2023) 28:947–61. 10.1037/met0000479 35113632

[27] Xiong Y Li H Yu M Deng Y Zhang Q Li S. Demoralization and depression in breast cancer: a Bayesian network and computer-simulated intervention study. Eur J Oncol Nurs. (2025) 78:102938. 10.1016/j.ejon.2025.102938 40714677

[28] Liu B Liu L Ren L Ma Z Zou M Li Y. Elucidating the relationship between burnout and sleep disturbances among firefighters: a network analysis. BMC Public Health. (2025) 25:240. 10.1186/s12889-025-21357-8 39833771 PMC11748853

[29] Gao Y Lin X Shen X Zhao X Zhao D Li P. Network analysis of fear of progression, anxiety, and depression in parents of children with malignant solid tumors: a cross-sectional study. J Psychosom Res. (2026) 202:112516. 10.1016/j.jpsychores.2025.112516 41494504

[30] Scutari M. Learning Bayesian networks with the bnlearn R package. J Stat Softw. (2010) 35:1–22. 10.18637/jss.v035.i0321603108

[31] Wu Y Ning X Zhong Q Li J Zhang H Li M. Relationships among neurocognition, social cognition, anxiety, and depression in patients with mild cognitive impairment: a network analysis. Am J Geriatr Psychiatry. (2026) 34:936–50. 10.1016/j.jagp.2025.10.008 41266238

[32] Jiang J Cheong AT Sazlina SG Lee K Chu D Yang L. Levels of active ageing and its influencing factors among older adults: a scoping review. BMC Public Health. (2026) 26:493. 10.1186/s12889-025-25820-4 41514282 PMC12882181

[33] Li C. Mapping the relationship between perceived involution and subjective well-being in Chinese university students: a network analysis across gender and grade levels. Front Psychol. (2025) 16:0–11. 10.3389/fpsyg.2025.1628064 PMC12713321 41426384

[34] Tüfekçi H Akansel N Sivrikaya SK. Pain interference with daily living activities and dependency level of patients undergoing CABG surgery. Pain Manag Nurs. (2022) 23:180–7. 10.1016/j.pmn.2021.03.002 33839038

[35] Ünver S Yildirim M Akbal S Sever S. Challenges experienced by cardiac intensive care nurses during first out-of-bed patient mobilization after open-heart surgery: a descriptive phenomenological qualitative study. J Adv Nurs. (2024) 80:4616–28. 10.1111/jan.16091 38318643

[36] Gülşen M Arslan S Ceren E Binokay H Doğan ÖF. The effect of pain on fear of movement in the early period after open heart surgery according to the fear-avoidance model: the role of moderating factors. BMC Nurs. (2025) 24:1413. 10.1186/s12912-025-04059-9 PMC12629076 41257859

[37] Li L Piao JJ Shang Z. Fatigue and physical activity in post-CABG patients: the parallel mediation model of kinesiophobia and self-efficacy : a prospective multicentre and cross-sectional study in China. BMJ Open. (2024) 14:e082576. 10.1136/bmjopen-2023-082576 38951011 PMC11328647

[38] Koenders N Van Oorsouw R Conijn D Verbakel M Eijsvogels TMH Van Der Wees PJ. Patient needs regarding cardiac rehabilitation: a systematic review and meta-ethnographic synthesis. J Am Heart Assoc. (2025) 14:e040588. 10.1161/JAHA.124.040588 40932109 PMC12554424

[39] Ibrahim D Elkhidir IH Mohammed Z Abdalla D’ Mohammed OA Hemmeda L. The role of CBT in enhancing health outcomes in coronary artery bypass graft patients: a systematic review. BMC Psychiatry. (2025) 25:22. 10.1186/s12888-024-06457-w 39773615 PMC11708169

[40] Murphy B Le Grande M Alvarenga M Worcester M Jackson A. Anxiety and depression after a cardiac event: prevalence and predictors. Front Psychol. (2020) 10:3010. 10.3389/fpsyg.2019.03010 32063868 PMC7000459

[41] Gu Y Wu X Chai Y Lin P Gao X Wang Y. Network analysis identifies core fear symptoms as screening clues for post-PCI rehabilitation in patients with acute myocardial infarction. Front Cardiovasc Med. (2026) 13:1805006. 10.3389/fcvm.2026.1805006 42375850 PMC13310681

[42] Chaudhury S Saini R Bakhla AK Singh J. Depression and anxiety following coronary artery bypass graft: current Indian scenario. Cardiol Res Pract. (2016) 2016:1–6. 10.1155/2016/2345184 PMC4789419 27034884

[43] Zeng Z Wan L Zheng J Shen Y Luo H He M. Summary of the best evidence for the management of kinesiophobia in patients after cardiac surgery. BMC Cardiovasc Disord. (2025) 25:127. 10.1186/s12872-025-04570-6 39994522 PMC11849301

[44] Tulloch H Greenman PS Tassé V. Post-traumatic stress disorder among cardiac patients: prevalence, risk factors, and considerations for assessment and treatment. Behav Sci. (2015) 5:27–40. 10.3390/bs5010027 PMC4384060 25545708

[45] Knotzer H Dünser MW Mayr AJ Hasibeder WR. Postbypass arrhythmias: pathophysiology, prevention, and therapy. Curr Opin Crit Care. (2004) 10:330–5. 10.1097/01.ccx.0000135512.18753.bc 15385747

[46] Marsoner K Voetsch A Lierzer C Sodeck GH Fruhwald S Dapunt O. Gastrointestinal complications following on-pump cardiac surgery—a propensity matched analysis. PLoS One. (2019) 14:e0217874. 10.1371/journal.pone.0217874 31166962 PMC6550404

[47] Narita M Tsutsui M Mochizuki N Suzuki F Setogawa Y Hirofuji A. Constipation in patients undergoing cardiac surgery: incidence and risk factors. JTCVS OPEN. (2025) 28:387–96. 10.1016/j.xjon.2025.08.007 41466787 PMC12745003

[48] Jarrah MI Hweidi IM Al-Dolat SA Alhawatmeh HN Al-Obeisat SM Hweidi LI. The effect of slow deep breathing relaxation exercise on pain levels during and post chest tube removal after coronary artery bypass graft surgery. Int J Nurs Sci. (2022) 9:155–61. 10.1016/j.ijnss.2022.03.001 35509699 PMC9052258

[49] Lahtinen P Kokki H Hynynen M. Pain after cardiac surgery: a prospective cohort study of 1-year incidence and intensity. Anesthesiology. (2006) 105:794–800. 10.1097/00000542-200610000-00026 17006079

[50] Öğüt S Sucu Dağ G. Pain characteristics and pain interference among patients undergoing open cardiac surgery. J Perianesth Nurs. (2019) 34:757–66. 10.1016/j.jopan.2018.10.009 30773408

[51] Zhou Y Gao W Gao S Guo X Liu M Cao C. Pain catastrophizing, kinesiophobia and exercise adherence in patients after total knee arthroplasty: the mediating role of exercise self-efficacy. J Pain Res. (2023) 16:3993–4004. 10.2147/JPR.S432106 38026453 PMC10676101

[52] Wynne R Jedwab RM Gjeilo KH Fredericks S Magboo R Phillips EK. A systematic review of multimodal analgesic effectiveness on acute postoperative pain after adult cardiac surgery. J Adv Nurs. (2025) 81:2757–92. 10.1111/jan.16688 39704393

[53] McDonall J Wilson J Botti M Hutchinson A. Patient experience of pain management following cardiac surgery: a mixed methods study. Pain Manag Nurs. (2025) 26:271–81. 10.1016/j.pmn.2024.10.012 39542767

[54] Li Y Jia S Cao B Chen L Shi Z Zhang H. Network analysis of somatic symptoms in Chinese patients with depressive disorder. Front Public Health. (2023) 11:1079873. 10.3389/fpubh.2023.1079873 36992877 PMC10040552

[55] Kluszczyńska M Młynarska A Mikulakova W. Influence of frailty syndrome on kinesiophobia according to the gender of patients after coronary artery bypass surgery. Health Care (Don Mills). (2021) 9:730. 10.3390/healthcare9060730 PMC8231869 34198555

