# Supplementary material for: Network analysis of kinesiophobia and fear of progression and their association with cardiac rehabilitation adherence in post-CABG patients
Source: Front Cardiovasc Med. 2026 Sep 29;13:1902087. doi: 10.3389/fcvm.2026.1902087 (PMC13657925; doi:10.3389/fcvm.2026.1902087)
Supplement: Supplementary file 1 [file Datasheet1.docx]

Supplementary Material

Table S1 Edge weights in the network.

| Edge (From / To) | | *Edge weight* |
| --- | --- | --- |
| FOP4 | FOP12 | 0.747016226589778 |
| FOP7 | FOP8 | 0.64770136705173 |
| FOA1 | FOA2 | 0.446007539205981 |
| FOA18 | FOA20 | 0.4444202228998 |
| FOP9 | FOP10 | 0.401366187665623 |
| FOA3 | FOA7 | 0.384076585536511 |
| FOA19 | FOA21 | 0.302530260190962 |
| FOP3 | FOP5 | 0.301574653849366 |
| FOP1 | FOP2 | 0.265829646827313 |
| FOA10 | FOA20 | 0.210084791621853 |
| FOA8 | FOA9 | 0.187935337755712 |
| FOA1 | FOA9 | 0.187820933289668 |
| FOA14 | FOA16 | 0.187669527786314 |
| FOP10 | FOP11 | 0.185004503221828 |
| FOA2 | FOA4 | 0.183007044348732 |
| FOA5 | FOA9 | 0.175776115014096 |
| FOP8 | FOP11 | 0.173160061655796 |
| FOP7 | FOP11 | 0.161331538684762 |
| FOA4 | FOA5 | 0.160339242553954 |
| FOA7 | FOA15 | 0.155550391454466 |
| FOA1 | FOA17 | 0.147012893303401 |
| FOP3 | FOP10 | 0.143027324553172 |
| FOA4 | FOA6 | 0.140740285055046 |
| FOP6 | FOP7 | 0.130423629462294 |
| FOA5 | FOA17 | 0.128199511719282 |
| FOA6 | FOA10 | 0.123895699060463 |
| FOA14 | FOA18 | 0.121255544621998 |
| FOP1 | FOP3 | 0.115383624116024 |
| FOA7 | FOA12 | 0.109145123795349 |
| FOA16 | FOA17 | 0.106616813357847 |
| FOA16 | FOA19 | 0.105422440794465 |
| FOA10 | FOA21 | 0.103376359391227 |
| FOA10 | FOA18 | 0.101030993024978 |
| FOA5 | FOA12 | 0.0989284361850623 |
| FOA2 | FOA3 | 0.0976708395720682 |
| FOA1 | FOA13 | 0.0967135039006418 |
| FOA6 | FOA16 | 0.0942541689193699 |
| FOA4 | FOA10 | 0.0942364986957795 |
| FOA3 | FOA5 | 0.0925399493041888 |
| FOA4 | FOP9 | 0.092097432719773 |
| FOA3 | FOP5 | 0.0890882630305947 |
| FOA4 | FOA8 | 0.0885896411243515 |
| FOP3 | FOP9 | 0.0880228243331908 |
| FOP1 | FOP6 | 0.0868118918179204 |
| FOA13 | FOA16 | 0.0864690038661455 |
| FOP9 | FOP11 | 0.0853021742534506 |
| FOA8 | FOA21 | 0.0850603823927503 |
| FOP1 | FOP11 | 0.0837640648859093 |
| FOA5 | FOA6 | 0.083727577661742 |
| FOP5 | FOP10 | 0.0827791944684275 |
| FOA2 | FOA6 | 0.082380660181142 |
| FOA3 | FOA12 | 0.0807086621823909 |
| FOA3 | FOA8 | 0.0806029216987943 |
| FOA6 | FOA8 | 0.0805374928373609 |
| FOA8 | FOA15 | 0.0802612960742762 |
| FOA4 | FOA9 | 0.0801753539307242 |
| FOP1 | FOP10 | 0.0792822173846634 |
| FOA15 | FOA18 | 0.0781602144410364 |
| FOP11 | FOP12 | 0.0777663816850667 |
| FOA20 | FOP1 | 0.077145699740473 |
| FOA16 | FOA21 | 0.0769322278350894 |
| FOA4 | FOA21 | 0.0764595211990034 |
| FOA15 | FOP5 | 0.0762130907466008 |
| FOA11 | FOP4 | -0.076180583 |
| FOP5 | FOP6 | 0.0754411365055886 |
| FOA6 | FOP6 | 0.0753990498252943 |
| FOA4 | FOA13 | 0.0751269365631154 |
| FOA7 | FOA8 | 0.0750114486626024 |
| FOA2 | FOA13 | 0.0735348617079893 |
| FOA7 | FOA10 | 0.0725413002622822 |
| FOP1 | FOP5 | 0.0699820593202584 |
| FOA4 | FOA14 | 0.0695131131258834 |
| FOP5 | FOP11 | 0.0686427354674164 |
| FOA12 | FOP8 | 0.0682070007884356 |
| FOA12 | FOA13 | 0.0680781853877572 |
| FOA7 | FOP3 | 0.0680286934474848 |
| FOA7 | FOP5 | 0.0668260370116684 |
| FOA17 | FOA19 | 0.0659788790633219 |
| FOA8 | FOP2 | 0.0654941457023295 |
| FOA6 | FOA18 | 0.0642850374449154 |
| FOA17 | FOA21 | 0.0641195271121654 |
| FOA5 | FOA16 | 0.062455595605741 |
| FOA3 | FOA11 | -0.061590004 |
| FOA9 | FOA14 | 0.0608773654498138 |
| FOA12 | FOP3 | 0.0588499031703739 |
| FOA8 | FOA20 | 0.0585590909598915 |
| FOA15 | FOP2 | 0.0581756480477304 |
| FOA10 | FOA15 | 0.0580075474496355 |
| FOA4 | FOA18 | 0.0569340082400505 |
| FOP2 | FOP3 | 0.0561002909378302 |
| FOA5 | FOA10 | 0.0556610355639402 |
| FOA20 | FOP5 | 0.0556421223837602 |
| FOA6 | FOP3 | 0.0545701225926205 |
| FOA3 | FOA18 | 0.0543821807340331 |
| FOA14 | FOP1 | 0.054131705142189 |
| FOP5 | FOP7 | 0.053215380030198 |
| FOA9 | FOA18 | 0.0528234491368412 |
| FOA6 | FOA13 | 0.051913476431377 |
| FOA5 | FOA21 | 0.0518392039314842 |
| FOA8 | FOA10 | 0.0517589166321788 |
| FOA9 | FOA15 | 0.0517146833624515 |
| FOA6 | FOP4 | 0.0515232171682215 |
| FOA9 | FOA13 | 0.0511384899117295 |
| FOA14 | FOP6 | 0.0487699368647406 |
| FOP8 | FOP12 | 0.0475930474345618 |
| FOP6 | FOP10 | 0.0475663132790334 |
| FOA2 | FOA16 | 0.0457536970679923 |
| FOA15 | FOA20 | 0.0450509854083544 |
| FOA12 | FOA14 | 0.0447000970616806 |
| FOP8 | FOP10 | 0.0444541459090849 |
| FOA5 | FOP3 | 0.0437395724280963 |
| FOA16 | FOP8 | 0.0431285588366524 |
| FOP6 | FOP8 | 0.0430794316598923 |
| FOA3 | FOP9 | 0.042624462916699 |
| FOA9 | FOP1 | 0.0425326682909116 |
| FOP2 | FOP12 | 0.0419597459269013 |
| FOP2 | FOP11 | 0.0412265859467395 |
| FOA11 | FOA20 | -0.041137257 |
| FOP2 | FOP5 | 0.0410003774500381 |
| FOA12 | FOP9 | 0.0402455255260609 |
| FOA10 | FOP2 | 0.0398600553799639 |
| FOA10 | FOA12 | 0.0393795479743495 |
| FOA2 | FOA17 | 0.0390870996421102 |
| FOA14 | FOP11 | 0.0382216952447782 |
| FOA4 | FOA15 | 0.0377594203917169 |
| FOP4 | FOP11 | 0.0375739624435523 |
| FOA14 | FOP3 | 0.0373231794894653 |
| FOA4 | FOA17 | 0.0372856003761154 |
| FOA15 | FOP3 | 0.0370576268231501 |
| FOA17 | FOP3 | -0.035936717 |
| FOP2 | FOP10 | 0.0358379409294468 |
| FOP1 | FOP12 | 0.0353639360899864 |
| FOA8 | FOP10 | 0.0351593087443606 |
| FOA16 | FOP2 | 0.0341993419901028 |
| FOP7 | FOP9 | 0.0334805841081216 |
| FOP4 | FOP5 | 0.0330776416143393 |
| FOA2 | FOA20 | 0.0328115961945655 |
| FOA8 | FOA18 | 0.032225307761877 |
| FOA1 | FOA4 | 0.031972319564878 |
| FOA6 | FOP11 | 0.0313816795454912 |
| FOA14 | FOP10 | 0.030826102013588 |
| FOA10 | FOA16 | 0.0296247203621259 |
| FOA12 | FOA15 | 0.0289270191417895 |
| FOA9 | FOA16 | 0.0286898436038966 |
| FOA10 | FOA14 | 0.0283740603337286 |
| FOA9 | FOP4 | -0.028362928 |
| FOA8 | FOP1 | 0.0283416577427925 |
| FOA9 | FOA21 | 0.028162931834768 |
| FOP6 | FOP9 | 0.0275923639231391 |
| FOP2 | FOP8 | 0.0274249216753933 |
| FOA4 | FOA16 | 0.0271948088558546 |
| FOA20 | FOP4 | 0.0270333905548764 |
| FOA11 | FOA19 | -0.026654492 |
| FOA3 | FOA17 | -0.026481942 |
| FOA13 | FOP4 | 0.0244710679093606 |
| FOA11 | FOA16 | -0.024084649 |
| FOA14 | FOP7 | 0.023519142712808 |
| FOA13 | FOA21 | 0.0234954604462569 |
| FOA11 | FOA14 | -0.023396884 |
| FOA6 | FOP5 | 0.0228708454348368 |
| FOA6 | FOA14 | 0.0221978216344393 |
| FOA4 | FOP7 | 0.0220437965454168 |
| FOA1 | FOP9 | 0.0218223639669604 |
| FOA9 | FOP2 | 0.021764196150528 |
| FOP8 | FOP9 | 0.0216335249347948 |
| FOA17 | FOP8 | 0.0216232460717203 |
| FOA4 | FOP3 | 0.0204901608259653 |
| FOA5 | FOA20 | 0.020340356896738 |
| FOA7 | FOA18 | 0.0201794049633418 |
| FOA6 | FOA7 | 0.0180730720496048 |
| FOA9 | FOA12 | 0.0172172189803894 |
| FOP9 | FOP12 | 0.0170250919252367 |
| FOA9 | FOP12 | -0.016660718 |
| FOA17 | FOP12 | 0.0162270203265927 |
| FOA20 | FOP6 | 0.0155083132085214 |
| FOA6 | FOP7 | 0.0154107468244804 |
| FOA3 | FOP3 | 0.0147107048696882 |
| FOA4 | FOP6 | 0.0146768628626211 |
| FOA11 | FOP12 | -0.014491899 |
| FOA8 | FOP12 | -0.014482736 |
| FOP1 | FOP4 | 0.0139858777731944 |
| FOA2 | FOA8 | 0.0136856684854937 |
| FOA6 | FOP1 | 0.0133896555464485 |
| FOA17 | FOP2 | 0.0132895589718288 |
| FOA14 | FOP9 | 0.0129209438999218 |
| FOA1 | FOA5 | 0.0124962123579524 |
| FOA20 | FOP3 | 0.0123021128916103 |
| FOA2 | FOA5 | 0.0119061908955858 |
| FOA8 | FOA14 | 0.0118547486080081 |
| FOA5 | FOP7 | 0.0115446587369894 |
| FOP3 | FOP4 | 0.0115139770266406 |
| FOA16 | FOP7 | 0.0113788348312276 |
| FOA2 | FOA14 | 0.0111984990934893 |
| FOA21 | FOP5 | 0.0108999238603637 |
| FOA1 | FOA16 | 0.0107338174070665 |
| FOA10 | FOP1 | 0.0106057994721335 |
| FOA10 | FOP5 | 0.0103988009491633 |
| FOA10 | FOP3 | 0.00983931879698944 |
| FOA4 | FOA19 | 0.00958041825042624 |
| FOA6 | FOA11 | -0.009548493 |
| FOA11 | FOP10 | -0.009148866 |
| FOA1 | FOA8 | 0.00905904904657832 |
| FOA8 | FOP11 | 0.0090002322998757 |
| FOA4 | FOA20 | 0.00855819486672092 |
| FOA5 | FOA8 | 0.00849059402764762 |
| FOA2 | FOA21 | 0.00834536006092934 |
| FOA9 | FOP8 | 0.00827818232678205 |
| FOA21 | FOP3 | 0.00761179497923862 |
| FOA4 | FOA7 | 0.00736040423290711 |
| FOA5 | FOA7 | 0.00691130121325464 |
| FOA6 | FOP9 | 0.0068769777441068 |
| FOP1 | FOP8 | 0.00652111629137256 |
| FOA8 | FOP4 | -0.005589969 |
| FOA10 | FOP6 | 0.00556955687597818 |
| FOA18 | FOP3 | 0.00520521988578419 |
| FOA5 | FOP2 | 0.00510106209760631 |
| FOA12 | FOA16 | 0.00488842422990452 |
| FOA2 | FOA9 | 0.00477538979660036 |
| FOA9 | FOA11 | -0.0043911 |
| FOA1 | FOA12 | 0.00384836197707381 |
| FOA10 | FOA19 | 0.00362816810112878 |
| FOA1 | FOA11 | 0.00360001187781997 |
| FOA16 | FOP4 | 0.00323408123500006 |
| FOA11 | FOP9 | -0.003048361 |
| FOP5 | FOP12 | 0.00302032159577396 |
| FOA12 | FOP2 | 0.00264731141517673 |
| FOA3 | FOA20 | 0.00256554047685265 |
| FOA8 | FOA19 | 0.00244146516555252 |
| FOA1 | FOA7 | 0.00221199209193404 |
| FOA16 | FOP1 | 0.00156519108469091 |
| FOA21 | FOP6 | 0.00109128789667924 |
| FOA13 | FOP7 | 0.00086820042816026 |
| FOA11 | FOP8 | -0.000254652 |

Table S2 Weight value in the Bayesian network model represented by each arrow.

| Edge direction | BIC Value | Direction Probability |
| --- | --- | --- |
| FOP8 -> FOP7 | -239.7684712 | 0.511 |
| FOP4 -> FOP12 | -156.5979038 | 0.5065 |
| FOA2 -> FOA1 | -110.1979666 | 0.6085 |
| FOP11 -> FOP10 | -101.1276917 | 0.52760736196319 |
| FOA20 -> FOA18 | -97.64724297 | 0.5355 |
| FOA4 -> FOA2 | -86.47665712 | 0.698469387755102 |
| FOA4 -> FOA6 | -85.88745109 | 0.70855614973262 |
| FOP10 -> FOP9 | -80.85003163 | 0.579 |
| FOP1 -> FOP2 | -70.5009803 | 0.727955911823647 |
| FOP3 -> FOP5 | -69.97333817 | 0.634769539078156 |
| FOA3 -> FOA7 | -46.47964507 | 0.51 |
| FOP7 -> FOP6 | -44.91083898 | 0.934113300492611 |
| FOA4 -> FOA9 | -27.56633945 | 0.875515818431912 |
| FOP5 -> FOA3 | -25.97897823 | 0.777403035413153 |
| FOA20 -> FOA10 | -24.94796881 | 0.576131687242798 |
| FOA14 -> FOA16 | -24.56818257 | 0.59703947368421 |
| FOP7 -> FOP5 | -22.39913802 | 0.78789592760181 |
| FOP10 -> FOP3 | -20.79598768 | 0.667377398720682 |
| FOA21 -> FOA16 | -20.7452103 | 0.548984468339307 |
| FOP11 -> FOP4 | -20.32691681 | 0.825331125827815 |
| FOA2 -> FOA3 | -18.19628858 | 0.664473684210526 |
| FOA4 -> FOA14 | -18.11080824 | 0.940257352941177 |
| FOA4 -> FOA5 | -16.56844726 | 0.867213114754098 |
| FOA1 -> FOA9 | -16.47469263 | 0.597308488612836 |
| FOA21 -> FOA19 | -13.35615611 | 0.906813627254509 |
| FOA9 -> FOA5 | -10.89631322 | 0.504797441364606 |
| FOA18 -> FOA14 | -10.89172614 | 0.702836004932182 |
| FOA4 -> FOA21 | -10.3799777 | 0.922106824925816 |
| FOP8 -> FOP11 | -10.25932177 | 0.572836801752464 |
| FOA9 -> FOA18 | -10.24731184 | 0.539967373572594 |
| FOP8 -> FOP12 | -9.055472131 | 0.875 |
| FOP8 -> FOA12 | -8.955376351 | 0.885220125786164 |
| FOA4 -> FOP9 | -8.699283498 | 0.569444444444444 |
| FOA9 -> FOA8 | -7.971305153 | 0.598998887652948 |
| FOA4 -> FOA10 | -7.767138574 | 0.714854111405836 |
| FOP7 -> FOP11 | -7.565930844 | 0.560406698564593 |
| FOA1 -> FOA17 | -7.302920635 | 0.825980392156863 |
| FOP9 -> FOP3 | -7.127274274 | 0.51043219076006 |
| FOP4 -> FOA11 | -6.652627333 | 0.611994219653179 |
| FOA6 -> FOA10 | -6.391428355 | 0.580487804878049 |
| FOP3 -> FOP1 | -6.36475198 | 0.813368055555555 |
| FOP5 -> FOP1 | -6.00159517 | 0.765567765567766 |
| FOA20 -> FOP1 | -5.752073111 | 0.600682593856655 |
| FOA3 -> FOA5 | -5.2952337 | 0.524752475247525 |
| FOA16 -> FOA17 | -5.179111647 | 0.646915584415584 |
| FOA7 -> FOA15 | -5.049083833 | 0.70986920332937 |
| FOA10 -> FOA21 | -4.556542183 | 0.795454545454546 |
| FOP5 -> FOA15 | -4.372014853 | 0.789437585733882 |
| FOA1 -> FOA13 | -4.033404295 | 0.840949033391916 |
| FOA7 -> FOA12 | -3.966168869 | 0.861821086261981 |
| FOA5 -> FOA17 | -3.915365013 | 0.687290969899666 |
| FOA18 -> FOA15 | -3.328723988 | 0.770192307692308 |
| FOA4 -> FOA8 | -3.27266211 | 0.913841807909605 |
| FOA20 -> FOA8 | -2.28645089 | 0.677215189873418 |
| FOA3 -> FOA12 | -2.211920351 | 0.814814814814815 |
| FOA6 -> FOP4 | -2.112631279 | 0.727678571428572 |
| FOP3 -> FOA7 | -2.062883596 | 0.756042296072508 |
| FOP3 -> FOA17 | -1.878324639 | 0.835016835016835 |
| FOA16 -> FOA19 | -1.678889844 | 0.720108695652174 |
| FOA9 -> FOA15 | -1.59709761 | 0.748245614035088 |
| FOA4 -> FOA13 | -1.286227658 | 0.878048780487805 |
| FOA7 -> FOA8 | -0.89212236 | 0.56544901065449 |
| FOA6 -> FOA13 | -0.872199608 | 0.762250453720508 |
| FOA3 -> FOA8 | 0.69733047420965 | 0.555984555984556 |
| FOP4 -> FOA9 | 0.473643629395724 | 0.541062801932367 |
| FOP5 -> FOA7 | 0.175516567097702 | 0.682310469314079 |

**Table S3** Edge weights between symptoms and cardiac rehabilitation adherence.

| Node | Weight |
| --- | --- |
| FOP3 | -0.158468191 |
| FOA2 | -0.109226416 |
| FOA4 | -0.083755422 |
| FOP6 | -0.082682303 |
| FOA5 | -0.076385565 |
| FOA18 | -0.05855554 |
| FOA14 | -0.054730707 |
| FOP1 | -0.045204969 |
| FOA8 | -0.044033381 |
| FOP11 | -0.043479973 |
| FOA21 | -0.043209347 |
| FOP10 | -0.03628243 |
| FOP4 | -0.022657907 |
| FOA17 | -0.014845087 |
| FOA15 | -0.014554824 |
| FOA19 | -0.009830861 |
| FOP5 | -0.007989829 |
| FOA1 | -0.006864222 |
| FOA9 | -0.004926741 |
| FOA3 | 0 |
| FOA6 | 0 |
| FOA7 | 0 |
| FOA10 | 0 |
| FOA11 | 0 |
| FOA12 | 0 |
| FOA13 | 0 |
| FOA16 | 0 |
| FOA20 | 0 |
| FOP2 | 0 |
| FOP7 | 0 |
| FOP8 | 0 |
| FOP9 | 0 |
| FOP12 | 0 |

**Supplementary Figure 1** Bootstrapped 95%CIs of estimated edge weights.

**Supplementary Figure 2** Stability of centrality indices by case dropping subset bootstrap.

**Supplementary Figure 3** Centrality difference test based on non-parametric bootstrapping. Black cells indicate statistically significant differences between centrality indices; gray cells indicate non-significant differences.

**Supplementary Figure 4** Edge-weight difference test based on non-parametric bootstrapping. Black cells indicate statistically significant differences between edge weights; gray cells indicate non-significant differences.

**Analysis code**

\# === 0. Load Required Packages ===

library(glmnet)

library(qgraph)

library(networktools)

library(ggplot2)

library(bootnet)

library(mgm)

library(dplyr)

library(tidyr)

library(NetworkComparisonTest)

\# Set global random seed for reproducibility

set.seed(12345)

\# === 1. Data Reading and Preprocessing ===

\# Read data

raw_data <- read.csv(".csv", check.names = FALSE)

\# Define variable names

tsk_vars <- paste0("FOA", 1:21) # Fear of activity (21 items)

fop_vars <- paste0("FOP", 1:12) # Fear of progression (12 items)

adh_var <- "ADH"	# Adherence (Target variable)

all_vars <- c(tsk_vars, fop_vars, adh_var)

\# Validate dataset columns

if (!all(all_vars %in% colnames(raw_data))) {

stop("Missing required columns in the dataset. Please check the CSV file.")

}

if (!"OLD" %in% colnames(raw_data)) {

stop("Missing grouping variable 'OLD' in the dataset.")

}

\# Extract and clean data (handle missing values)

full_data <- raw_data[, c("OLD", all_vars)]

full_data <- na.omit(full_data)

\# Define groups and color palettes

groups_list <- list("Fear of activity" = 1:21, "Fear of progression" = 22:33)

my_colors	<- c("#87CEFA", "#FF6B6B")

groups_vec <- c(rep('FOA', 21), rep('FOP', 12))

labels_sym <- c(tsk_vars, fop_vars)

\# ==============================================================================

\# Part 1: Overall Network Analysis (TSK & FOP, Excluding ADH)

\# ==============================================================================

cat("\n--- Running Part 1: Overall Network Analysis ---\n")

symptom_data <- as.data.frame(full_data[, c(tsk_vars, fop_vars)])

labels <- colnames(symptom_data)

groups <- groups_vec

\# 1. Network Estimation

set.seed(12345)

network1 <- estimateNetwork(

symptom_data,

default = "EBICglasso",

tuning = 0.5,

corMethod = "cor",

corArgs = list(method = "spearman", use = "pairwise.complete.obs")

)

\# 2. Predictability Calculation (MGM)

set.seed(12345)

data_matrix <- as.matrix(symptom_data)

mgm_fit <- mgm(

data = data_matrix,

type = rep("g", 33),

level = rep(1, 33),

lambdaSel = "EBIC",

lambdaGam = 0.5,

binarySign = TRUE

)

predictability_values <- predict(mgm_fit, data_matrix, errorCon = "R2")$errors[, "R2"]

\# Export predictability values

cat("\n--- Exporting Predictability Values (Part 1) ---\n")

if (exists("predictability_values") && exists("labels")) {

pred_df <- data.frame(

Node = labels,

Predictability_R2 = round(predictability_values, 4)

)

pred_df <- pred_df[order(pred_df$Predictability_R2, decreasing = TRUE), ]

print(pred_df)

write.csv(pred_df, "Node_Predictability_Part1.csv", row.names = FALSE)

cat("Node predictability values saved to 'Node_Predictability_Part1.csv'.\n")

}

\# 3. Plot Network Graph

tiff("Network_TSK_FOP.tiff", width = 3500, height = 3000, units = 'px', res = 600, compression = "lzw")

qgraph(

network1$graph, layout = "spring",

pie = predictability_values, pieColor = "red",

labels = labels,

groups = groups_list, color = my_colors,

legend.cex = 0.3,

GLratio = 2.5,

shape = "circle", vsize = 5, esize = 12, edge.labels = FALSE,

theme = "colorblind",

mar = c(5, 5, 5, 5)

)

dev.off()

cat("Network graph saved as 'Network_TSK_FOP.tiff'.\n")

\# 4. Export Edge Weights

weights_matrix <- network1$graph

rownames(weights_matrix) <- labels

colnames(weights_matrix) <- labels

edge_indices <- which(weights_matrix != 0 & upper.tri(weights_matrix), arr.ind = TRUE)

edge_data <- data.frame(

Node1 = labels[edge_indices[, 1]],

Node2 = labels[edge_indices[, 2]],

Weight = weights_matrix[edge_indices]

)

get_grp <- function(x) ifelse(grepl("TSK|FOA", x), "FOA", "FOP")

edge_data$Type <- ifelse(sapply(edge_data$Node1, get_grp) != sapply(edge_data$Node2, get_grp), "Bridge", "Within")

edge_data <- edge_data[order(abs(edge_data$Weight), decreasing = TRUE), ]

write.csv(edge_data, "TSK_FOP_All_Edges.csv", row.names = FALSE)

write.csv(edge_data[edge_data$Type == "Bridge", ], "TSK_FOP_Bridge_Edges.csv", row.names = FALSE)

\# ==============================================================================

\# Part 1.5: Centrality Analysis

\# ==============================================================================

cat("\n--- Generating and Saving Centrality Plots ---\n")

tiff("Centrality_Standard_TSK_FOP.tiff", width = 2500, height = 2500, units = 'px', res = 300, compression = "lzw")

centralityPlot(network1, include = c("ExpectedInfluence"), scale = "raw")

dev.off()

b <- bridge(network1$graph, communities = groups, directed = FALSE)

tiff("Centrality_Bridge_TSK_FOP.tiff", width = 2500, height = 2500, units = 'px', res = 300, compression = "lzw")

plot(b, include = c("Bridge Expected Influence (1-step)"),

theme_bw = FALSE, raw0 = TRUE, signed = TRUE)

dev.off()

cat("\n--- Extracting EI and BEI Values ---\n")

overall_ei <- colSums(network1$graph)

b <- bridge(network1$graph, communities = groups_vec, directed = FALSE)

overall_bei <- b$`Bridge Expected Influence (1-step)`

if (length(overall_ei) != 33 || length(overall_bei) != 33) {

stop("Length calculation error for EI or BEI (must equal 33).")

}

overall_metrics_df <- data.frame(

Node = names(overall_ei),

EI_Value = as.numeric(overall_ei),

BEI_Value = as.numeric(overall_bei)

)

overall_metrics_df <- overall_metrics_df[order(abs(overall_metrics_df$BEI_Value), decreasing = TRUE), ]

print(overall_metrics_df)

write.csv(overall_metrics_df, "Overall_EI_BEI_Specific_Values.csv", row.names = FALSE)

\# ==============================================================================

\# Part 1.6: Network Stability Analysis

\# ==============================================================================

cat("\n--- Running Stability Analysis ---\n")

set.seed(12345)

nonParametricBoot <- bootnet(

symptom_data, nBoots = 1000,

default = "EBICglasso", threshold = FALSE,

type = "nonparametric",

nCores = 1,

statistics = "all"

)

tiff("Stability_NonParametric_Edges.tiff", width = 2000, height = 1500, res = 300)

plot(nonParametricBoot, labels = FALSE, order = "sample")

dev.off()

tiff("Stability_Edge_Difference.tiff", width = 3000, height = 3000, units = 'px', res = 300, compression = "lzw")

plot(nonParametricBoot, plot = "difference", onlyNonZero = TRUE, order = "sample")

dev.off()

set.seed(12345)

centralityBoot <- bootnet(

symptom_data,

nBoots	= 1000,

default	= "EBICglasso",

threshold	= FALSE,

type	= "nonparametric",

nCores	= 1,

statistics = c("ExpectedInfluence", "BridgeExpectedInfluence"),

communities = groups

)

tiff("Stability_Centrality_Difference.tiff", width = 3000, height = 3000, units = 'px', res = 300, compression = "lzw")

plot(centralityBoot,

plot	= "difference",

statistics = c("ExpectedInfluence", "BridgeExpectedInfluence"),

order	= "sample")

dev.off()

set.seed(12345)

combo_boot <- bootnet(

symptom_data,

nBoots	= 1000,

default	= "EBICglasso",

threshold	= FALSE,

type	= "case",

nCores	= 1,

statistics = c("ExpectedInfluence", "BridgeExpectedInfluence"),

communities = groups,

fun	= ei_bridge_ei

)

tiff("Stability_Combo_EI_BridgeEI.tiff", width = 2000, height = 1500, res = 300)

plot(combo_boot,

statistics = c("ExpectedInfluence", "BridgeExpectedInfluence"),

plot	= "area",

xlab	= "Sample dropped",

ylab	= "Correlation with original",

main	= "Stability of EI and Bridge EI")

dev.off()

cs_results <- corStability(combo_boot)

cat("Expected Influence CS:", round(cs_results["expectedInfluence"], 3), "\n")

cat("Bridge Expected Influence CS:", round(cs_results["bridgeExpectedInfluence"], 3), "\n")

\# ==============================================================================

\# Part 2: Sample Size Simulation Analysis (netSimulator)

\# ==============================================================================

cat("\n--- Running Sample Size Simulation Analysis ---\n")

true_network <- network1$graph

test_n_cases <- c(250, 275, 300)

set.seed(12345)

simulation_results <- netSimulator(

input = true_network,

nCases = test_n_cases,

nReps = 100,

nCores = 1,

default = "EBICglasso",

tuning = 0.5,

corMethod = "cor",

corArgs = list(method = "spearman", use = "pairwise.complete.obs")

)

print(simulation_results)

png("Sample_Size_Simulation_Curve.png", width = 3000, height = 2000, res = 300)

plot(simulation_results)

dev.off()

\# ==============================================================================

\# Part 3: Flow Network Analysis (Including ADH)

\# ==============================================================================

cat("\n--- Running Part 2: Flow Network Analysis with ADH ---\n")

network_data_p2 <- as.data.frame(full_data[, c(tsk_vars, fop_vars, adh_var)])

labels_p2 <- colnames(network_data_p2)

results_adh <- estimateNetwork(

network_data_p2,

default = "EBICglasso",

tuning = 0.5,

corMethod = "cor",

corArgs = list(method = "spearman", use = "pairwise.complete.obs")

)

data_matrix_p2 <- as.matrix(network_data_p2)

mgm_fit_p2 <- mgm(

data = data_matrix_p2,

type = rep("g", 34),

level = rep(1, 34),

lambdaSel = "EBIC",

lambdaGam = 0.5,

binarySign = TRUE

)

predictability_values_p2 <- predict(mgm_fit_p2, data_matrix_p2, errorCon = "R2")$errors[, "R2"]

groups_list_p2 <- list(

"Fear of activity" = 1:21,

"Fear of progression" = 22:33,

"Adherence" = 34

)

my_colors_2 <- c("#87CEFA", "#FF6B6B", "#00BA38")

base_qgraph_object <- qgraph(

results_adh$graph,

layout = "spring",

DoNotPlot = TRUE,

pie = predictability_values_p2,

pieColor = "red",

labels = labels_p2,

groups = groups_list_p2,

color = my_colors_2,

shape = "circle",

vsize = 5,

esize = 12,

theme = "colorblind",

legend.cex = 0.3,

GLratio = 2,

posCol = "#0000FF",

negCol = "#FF0000",

mar = c(5, 5, 5, 4)

)

tiff("Flow_Network_ADH.tiff", width = 4500, height = 3000, units = 'px', res = 600, compression = "lzw")

flow(

base_qgraph_object,

from = "ADH",

horizontal = TRUE,

standardize = FALSE,

DoNotPlot = FALSE

)

dev.off()

weights_matrix <- results_adh$graph

rownames(weights_matrix) <- labels_p2

colnames(weights_matrix) <- labels_p2

if ("ADH" %in% rownames(weights_matrix)) {

adh_conn <- weights_matrix["ADH", ]

adh_conn <- adh_conn[names(adh_conn) != "ADH"]

res_df <- data.frame(Node = names(adh_conn), Weight = as.numeric(adh_conn))

res_df <- res_df[order(abs(res_df$Weight), decreasing = TRUE), ]

write.csv(res_df, "ADH_Connection_Strength.csv", row.names = FALSE)

}

\# ==============================================================================

\# Part 4: Comparative Network Analysis (Grouped by OLD)

\# ==============================================================================

cat("\n--- Running Part 3: Comparative Network Analysis by Group (OLD) ---\n")

data_low <- as.data.frame(full_data[full_data$OLD == 0, c(tsk_vars, fop_vars)])

data_high <- as.data.frame(full_data[full_data$OLD == 1, c(tsk_vars, fop_vars)])

cat("Low Age Group (OLD=0) Sample Size:", nrow(data_low), "\n")

cat("High Age Group (OLD=1) Sample Size:", nrow(data_high), "\n")

set.seed(12345)

net_low <- estimateNetwork(data_low, default = "EBICglasso", tuning = 0.5,

corMethod = "cor", corArgs = list(method = "spearman", use = "pairwise.complete.obs"))

set.seed(12345)

net_high <- estimateNetwork(data_high, default = "EBICglasso", tuning = 0.5,

corMethod = "cor", corArgs = list(method = "spearman", use = "pairwise.complete.obs"))

set.seed(12345)

mat_low <- as.matrix(data_low)

mgm_low <- mgm(data = mat_low, type = rep("g", 33), level = rep(1, 33),

lambdaSel = "EBIC", lambdaGam = 0.5, binarySign = TRUE, progressbar = FALSE)

pred_low <- predict(mgm_low, mat_low, errorCon = "R2")$errors[, "R2"]

set.seed(12345)

mat_high <- as.matrix(data_high)

mgm_high <- mgm(data = mat_high, type = rep("g", 33), level = rep(1, 33),

lambdaSel = "EBIC", lambdaGam = 0.5, binarySign = TRUE, progressbar = FALSE)

pred_high <- predict(mgm_high, mat_high, errorCon = "R2")$errors[, "R2"]

Layout_Avg <- averageLayout(net_low$graph, net_high$graph)

tiff("Network_Comparison_Age.tiff", width = 6500, height = 3000, units = 'px', res = 300, compression = "lzw")

par(mfrow = c(1, 2))

qgraph(net_low$graph, layout = Layout_Avg,

pie = pred_low, pieColor = "red",

groups = groups_list, color = my_colors,

labels = labels_sym,

title = "Middle-aged Group",

legend.cex = 0.3,

GLratio = 2.5,

shape = "circle", vsize = 5, esize = 12, edge.labels = FALSE,

theme = "colorblind",

mar = c(5, 5, 5, 5))

qgraph(net_high$graph, layout = Layout_Avg,

pie = pred_high, pieColor = "red",

groups = groups_list, color = my_colors,

labels = labels_sym,

title = "Older Group",

legend.cex = 0.3,

GLratio = 2.5,

shape = "circle", vsize = 5, esize = 12, edge.labels = FALSE,

theme = "colorblind",

mar = c(5, 5, 5, 5))

dev.off()

par(mfrow = c(1, 1))

\# Centrality metrics comparison & plotting

ei_low <- colSums(net_low$graph)

ei_high <- colSums(net_high$graph)

bei_low_res <- bridge(net_low$graph, communities = groups_vec, directed = FALSE)

bei_low <- bei_low_res$`Bridge Expected Influence (1-step)`

bei_high_res <- bridge(net_high$graph, communities = groups_vec, directed = FALSE)

bei_high <- bei_high_res$`Bridge Expected Influence (1-step)`

metrics_df <- data.frame(

Node = rep(labels_sym, 2),

Group = c(rep("Low Age", 33), rep("High Age", 33)),

EI = c(ei_low, ei_high),

BEI = c(bei_low, bei_high)

)

metrics_df$Node <- factor(metrics_df$Node, levels = labels_sym)

p_ei <- ggplot(metrics_df, aes(x = EI, y = Node, color = Group, group = Node)) +

geom_line(aes(group = Node), color = "gray80", size = 1) +

geom_point(size = 3) +

labs(x = "Expected Influence (EI)", y = "Symptoms", title = "Comparison of Expected Influence (EI)") +

theme_minimal() +

scale_color_manual(values = c("Low Age" = "#1f77b4", "High Age" = "#ff7f0e")) +

theme(legend.position = "top")

ggsave("Comparison_EI.tiff", p_ei, width = 8, height = 10, dpi = 300)

p_bei <- ggplot(metrics_df, aes(x = BEI, y = Node, color = Group, group = Node)) +

geom_line(aes(group = Node), color = "gray80", size = 1) +

geom_point(size = 3) +

labs(x = "Bridge Expected Influence (BEI)", y = "Symptoms", title = "Comparison of Bridge Expected Influence (BEI)") +

theme_minimal() +

scale_color_manual(values = c("Low Age" = "#1f77b4", "High Age" = "#ff7f0e")) +

theme(legend.position = "top")

ggsave("Comparison_BEI.tiff", p_bei, width = 8, height = 10, dpi = 300)

res_table <- metrics_df %>% pivot_wider(names_from = Group, values_from = c(EI, BEI))

write.csv(res_table, "Comparison_Metrics_Results.csv", row.names = FALSE)

\# Network Comparison Test (NCT)

set.seed(12345)

if (!exists("nct_res")) {

cat("\n--- Running Network Comparison Test (NCT) ---\n")

nct_res <- NCT(

data_low, data_high,

gamma = 0.5,

it = 1000,

binary.data = FALSE,

test.edges = TRUE,

edges = "all",

test.centrality = TRUE,

centrality = c("expectedInfluence"),

progressbar = TRUE

)

}

cat("\n=== NCT Test Results ===\n")

print(nct_res)

tiff("NCT_Results.tiff", width = 3000, height = 1500, units = 'px', res = 300)

par(mfrow = c(1, 2))

plot(nct_res, what = "network", main = "Network Invariance (Structure)")

plot(nct_res, what = "strength", main = "Global Strength Invariance")

dev.off()

cat("\n=== All Analysis Tasks Completed Successfully ===\n")

\# === 0. Load Required Packages ===

library(bnlearn)

library(qgraph)

library(igraph)

library(dplyr)

library(Rgraphviz)

\# === 1. Data Reading and Preprocessing ===

\# Read dataset

df <- read.csv(".csv")

df <- data.frame(lapply(df, as.numeric))

\# Define short names to match the 33 nodes (21 FOA items, 12 FOP items)

short_names <- c(

paste0("FOA", 1:21), # Fear of activity (21 nodes)

paste0("FOP", 1:12) # Fear of progression (12 nodes)

)

\# Ensure column names match short_names

colnames(df) <- short_names

\# ==============================================================================

\# Part 2: Structure Learning and Bootstrap Analysis

\# ==============================================================================

set.seed(12345)

boot <- boot.strength(df, R = 1000, algorithm = "hc", algorithm.args = list(score = "bic-g"))

\# Get optimal threshold

opt_threshold <- inclusion.threshold(boot)

cat("Optimal threshold applied:", opt_threshold, "\n")

\# Generate averaged network

avg_net <- averaged.network(boot, threshold = opt_threshold)

\# ==============================================================================

\# Part 3: Extracting Required Statistics (Edge Weights & BIC)

\# ==============================================================================

dag_net <- cextend(avg_net, strict = FALSE)

\# 1. Calculate total number of arcs (edges)

num_arcs <- narcs(dag_net)

cat("Total number of arcs in the network:", num_arcs, "\n")

\# 2. Calculate connection strength (Weight / BIC) between nodes

arc_weights <- arc.strength(dag_net, data = df, criterion = "bic-g")

\# 3. Format edge directions and weights into a structured data frame

final_edges <- merge(arc_weights, boot, by = c("from", "to"))

final_edges_formatted <- final_edges %>%

mutate(

Edge_direction = paste0(from, " -> ", to),

BIC_Value = strength.x

) %>%

select(Edge_direction, BIC_Value, Boot_Probability = strength.y, Direction_Probability = direction) %>%

arrange(desc(abs(BIC_Value))) # Sort by absolute BIC value in descending order

print("Node connection list and weights (BIC):")

print(final_edges_formatted)

\# 4. Export to CSV for manuscript tables

write.csv(final_edges_formatted, "", row.names = FALSE)

\# ==============================================================================

\# Part 4: Network Visualization and Figure Export

\# ==============================================================================

vars_foa <- short_names[1:21]

vars_fop <- short_names[22:33]

g_obj <- strength.plot(

avg_net, boot, shape = "ellipse", layout = "dot",

threshold = opt_threshold, render = FALSE

)

attrs <- list(

node = list(fontsize = 12, height = 0.5, fixedsize = FALSE),

graph = list(rankdir = "TB", nodesep = 0.5, ranksep = 0.5)

)

g_obj <- layoutGraph(g_obj, attrs = attrs, layoutType = "dot")

\# Set node fill colors (First 21 FOA nodes = light blue, Last 12 FOP nodes = red)

node_fill_colors <- c(

setNames(rep("#87CEFA", 21), names(df)[1:21]),

setNames(rep("#FF6B6B", 12), names(df)[22:33])

)

nodeRenderInfo(g_obj)$fill <- node_fill_colors[nodes(g_obj)]

nodeRenderInfo(g_obj)$col <- sapply(nodes(g_obj), function(x) "black")

\# Export high-resolution plot image

png(".png", height = 12, width = 18, units = "in", res = 500)

layout(matrix(c(1, 2), nrow = 1), widths = c(0.75, 0.25))

renderGraph(g_obj)

\# Draw legend

par(mar = c(0, 0, 0, 0))

plot(1, type = "n", axes = FALSE, xlab = "", ylab = "")

legend(

"center",

legend = c("Fear of activity", "Fear of progression"),

pt.bg = c("#87CEFA", "#FF6B6B"),

col	= "black",

pch	= 21,

pt.cex = 2.5,

cex	= 1.5,

bty	= "n",

y.intersp = 1.5

)

dev.off()

cat("\n=== All Bayesian Network Tasks Completed Successfully ===\n")
